# Low-Dose Bisphenol-A Impairs Adipogenesis and Generates Dysfunctional 3T3-L1 Adipocytes

**DOI:** 10.1371/journal.pone.0150762

**Published:** 2016-03-04

**Authors:** Fabiana Ariemma, Vittoria D’Esposito, Domenico Liguoro, Francesco Oriente, Serena Cabaro, Antonietta Liotti, Ilaria Cimmino, Michele Longo, Francesco Beguinot, Pietro Formisano, Rossella Valentino

**Affiliations:** 1 Department of Translational Medical Sciences, Federico II University of Naples, via S. Pansini, 5, 80131, Naples, Italy; 2 URT Genomic of Diabetes, Institute of Experimental Endocrinology and Oncology (IEOS), National Council of Research (CNR), via S. Pansini, 5, 80131, Naples, Italy; Universidad Miguel Hernández de Elche, SPAIN

## Abstract

Environmental endocrine disruptors (EDCs), including bisphenol-A (BPA), have been recently involved in obesity and diabetes by dysregulating adipose tissue function. Our aim was to examine whether prolonged exposure to low doses of BPA could affect adipogenesis and adipocyte metabolic functions. Therefore, 3T3-L1 pre-adipocytes were cultured for three weeks with BPA 1nM to mimic human environmental exposure. We evaluated BPA effect on cell proliferation, differentiation, gene expression and adipocyte metabolic function. BPA significantly increased pre-adipocyte proliferation (p*<*0.01). In 3T3-L1 adipocytes differentiated in the presence of BPA, the expression of Peroxisome proliferator-activated receptor gamma (PPARγ), Fatty Acid Binding Protein 4/Adipocyte Protein 2 (FABP4/AP2) and CCAAT/enhancer binding protein (C/EBPα) was increased by 3.5, 1.5 and 3 folds, respectively. Mature adipocytes also showed a significant increase in lipid accumulation (p*<*0.05) and alterations of insulin action, with significant reduction in insulin-stimulated glucose utilization (p*<*0.001). Moreover, in mature adipocytes, mRNA levels of Leptin, interleukin-6 (IL6) and interferon-γ (IFNγ) were significantly increased (p<0.05). In conclusion, BPA prolonged exposure at low doses, consistent with those found in the environment, may affect adipocyte differentiation program, enhancing pre-adipocyte proliferation and anticipating the expression of the master genes involved in lipid/glucose metabolism. The resulting adipocytes are hypertrophic, with impaired insulin signaling, reduced glucose utilization and increased pro-inflammatory cytokine expression. Thus, these data supported the hypothesis that BPA exposure, during critical stages of adipose tissue development, may cause adipocyte metabolic dysfunction and inflammation, thereby increasing the risk of developing obesity-related diseases.

## 1. Introduction

Obesity is a significant public health problem in the developed world, caused by complex interactions among genetic, behavioral and environmental factors [1]. Obesity is characterized by increased fat cell size (hypertrophic obesity) or cell number (hyperplasic obesity), often related to high caloric diet [2]. In the last few years, a role of environmental chemicals in promoting obesity has been suggested, with adverse effects on human health through adipose tissue dysfunction [3,4]. Among these chemical substances, endocrine disrupting compounds (EDCs) have a crucial role in mimicking hormonal activities, in promoting adipogenesis and in development of obesity and diabetes [3,5,6]. In particular, bisphenol-A (BPA), a xeno-estrogen plastic component, can be considered a “bona fide” adipogenic candidate, for its worldwide distribution and the capability to accumulate into and to affect adipose tissue [7–9].

Although BPA has short half-life, humans are chronically exposed to low doses of the compound, mainly through modern fast-food/processed/packaged food diet, dust and thermal paper. Indeed, BPA is able to leach from food and beverage containers upon heating or following pH changes [8,10]. Free and conjugated BPA are therefore measurable in human body fluids, such as blood, urine, saliva, amniotic fluid and breast milk [10,11].

Interestingly, in humans, BPA crosses the placenta, it is partially inactivated in fetal liver by uridine 5'-diphospho-glucuronosyl-transferase (UDP-UGT) enzyme and it accumulates in amniotic fluid and in adipose tissue, mainly as free form [12]. Considering that the embryonic and fetal life are periods of rapid cell division and epigenetic remodeling, BPA exposure in these critical windows can lead to permanent changes, by developmental “programming” metabolic dysfunctions later on in the lifespan, and contributing to insulin resistance and diabetes [13]. In animal studies, BPA, at concentrations within the human exposure range, may cause disruption of pancreatic beta-cell and anti-androgen effects with male infertility. In addition, for its interference on thyroid hormone receptor (TR), it may affect brain morphology and expression of genes related to brain development [7,10,14,15]. The impact of BPA on human health has been suspected in epidemiological and cross-sectional studies. Although these evidences are still controversial, a potential relationship between concurrent urinary/plasma BPA levels and obesity or obesity-related disorders was suggested [6,8,11,16,17].

Furthermore, BPA metabolic and inflammatory effects have also been evidenced in vitro on human and murine adipocytes, with decreased insulin action and increased pro-inflammatory cytokine secretion [9,18].

Thus, the possibility that BPA may affect adipose tissue function, contributing to the development of systemic low-grade inflammation, insulin resistance and metabolic syndrome, together with other environmental factors, such as high-fat/high calories diets, is to be highly considered [18–20]. However, the mechanisms by which BPA can interfere on adipocyte maturation and metabolic functions have been only partially elucidated.

Aim of this study was to investigate chronic BPA effect on adipogenesis, in term of master gene expression and on mature adipocyte function. For this purpose, in these experiments we used 3T3-L1 pre-adipocytes, a well established cell model of adipocyte differentiation.

## 2. Materials and Methods

### 2.1. Materials

BPA was dissolved in ethanol and was a generous gift of Prof. C. Crescenzi (Department of Pharmaceutical and Biomedical Science, University of Salerno, Fisciano- SA, Italy. For western blot analysis, antibodies against phospho-Ser473 Protein Kinase B (pPKB/AKT1), total PKB/AKT, total Extracellular Signal-Regulated Kinase (ERK1), Peroxisome proliferator-activated receptor gamma (PPARγ), Fatty Acid Binding Protein 4/Adipocyte protein 2 (FABP4/AP2) and 14-3-3 were purchased by Santa Cruz Biotechnology, Inc. (CA, USA). Antibody against Phospho-ERK1 (pERK1) was from Cell Signaling Technology, Inc. (Danvers, MA, USA) and antibody anti-Glucose transporter type 4 (GLUT-4) was from Abcam, Cambridge Science Park, Cambridge (CB4 0FW, UK). Horseradish peroxidase-conjugated secondary antibody at different concentrations was used.

All the other chemicals were from Sigma-Aldrich (St. Louis, USA, MO). Media and antibiotics for cell culture were from Lonza (Lonza Group Ltd, Basel, Switzerland), sera from Gibco (Gibco, CA, USA, 16010–159).

### 2.2. Cell culture, growth and differentiation

3T3-L1 mouse fibroblasts (ATCC CL-173) were available in host laboratory. 3T3-L1 were cultured in Dulbecco’s modified Eagle’s medium (DMEM) supplemented with 10% calf serum (CS) and 2% glutamine, 100 IU/ml penicillin and 100 IU/ml streptomycin. Cultures were maintained in humidified atmosphere of 95% air and 5% CO2 at 37°C. 3T3-L1 pre-adipocytes were treated with 1nM of BPA for three weeks before adipogenesis started and throughout the differentiation. Adipogenesis was performed as previously reported [21–24]. In brief, to verify the BPA effect on adipogenesis, 3T3-L1 pre-adipocytes were treated with BPA 1nM for two weeks. Next, at day 15 (the end of second week), they were seeded for cell growth determination in 6-well culture plates in complete medium [25] and counted after 24 (day 16), 48 (day 17) and 72 hours (days 18), using Burker chamber, according to the manufacturer’s instruction. To achieve adipocyte differentiation, the same number of cells were left to reach the confluence (day -2). Thereafter, untreated and BPA pre-treated murine fibroblasts were incubated with the first differentiation mix (DMEM 10% fetal bovine serum -FBS), containing insulin 174nM, dexamethasone 10mM and 3-isobutyl-1-methylxanthine 0.5mM for starting adipogenesis (day 0). Where indicated, BPA was included in the mix. Cells were collected and analyzed at days -2, 0, 4 and 8 during differentiation. In particular, at day 2 of adipogenesis the medium was replaced with the second differentiation mix (DMEM 10% FBS containing only insulin 174nM). Then, media were changed every two days (DMEM 10% FBS without insulin) [21, 26–29] until the mature adipocytes were obtained (day 8). All these mix changes were done in parallel in control cells without BPA.

### 2.3. Gene expression analysis using Real-time RT-PCR

To evaluate BPA effect on adipose tissue differentiation and function, mRNA expression of key adipogenic markers, transcription factors and cytokine production were assayed using Real-time RT-PCR. Total RNA was isolated from 3T3-L1 cells by using the Rneasy Kit (Qiagen, Valencia, CA, USA) according to the manufacturer's instruction and 1μg RNA was reverse-transcribed using SuperScript III Reverse Transcriptase (Life Technologies, Carlsbad, CA, USA). Quantitative real-time RT-PCR was performed with SYBR Green mix (Bio-Rad, Hercules, CA, USA) using an iCycler IQ multicolor Real-Time PCR Detection System (Bio-Rad, Hercules, CA, USA). All reactions were performed in triplicate and β-actin was used as an internal standard.

All primer sequences used were described in Table 1.

**Table 1:** Primer sequences used in Real-time RT-PCR analysis.

Primers	Sequences
C/EBPα	Forward 5’ – TGGACAAGAACAGCAACG – 3’
	Reverse 5’ – GTCAACTCCAGCACCTTC – 3’
FABP4/AP2	Forward 5’ – AATCACCGCAGACGACAG – 3’
	Reverse 5’ – ACGCCTTTCATAACACATTCC – 3’
PPARγ	Forward 5’ – TGGTGCCTTCGCTGATGC – 3’
	Reverse 5’ – CTGTGGTAAAGGGCTTGATGGCT – 3’
GLUT-4	Forward 5’ – TGCTCTCCGGTTCCGTGGGT – 3’
	Reverse 5’ – GGTTCCCCATCGTCAGAGCCG – 3’
GLUT-1	Forward 5’ – GGGAATGTCCTCATCTTGGA – 3’
	Reverse 5’ – TGAGGCTCTGTGTGGTTCTG – 3’
IL6	Forward 5’ – GGAGTGGCTAAGGACCAAGAC – 3’
	Reverse 5’ – GCATAACGCACTAGGTTTGCC – 3’
INFγ	Forward 5’ – GCTTTGCAGCTCTTCCTCAT – 3’
	Reverse 5’ – GTCACCATCCTTTTGCCAGT – 3’
Leptin	Forward 5’ – ACATTTCACACACGCAGTCG – 3’
	Reverse 5’ – GCATAACGCACTAGGTTTGCC – 3’
Adiponectin	Forward 5’ – GACGACACCAAAAGGGCTCA – 3’
	Reverse 5’ – GAGTGCCATCTCTGCCATCA – 3’
TNFα	Forward 5’ – AGCCCCCAGTCTGTATCCTT – 3’
	Reverse 5’ – CTCCCTTTGCAGAACTCAGG – 3’
β-actin	Forward 5’ – GGTGGGAATGGGTCAGAAGG – 3’
	Reverse 5’ – GTTGGCCTTAGGGTTCAGGG – 3’

### 2.4. Cell lysates and immunoblot procedure

Total cell lysates were obtained and separated by SDS-PAGE. Briefly, cells were solubilized for 20 min at 4 C with lysis buffer containing 50 mM HEPES, 150 mM NaCl, 10 mM EDTA, 10 mM Na_4_P_2_O_7_, 2 mM sodium orthovanadate, 50 mM NaF, 1 mM phenylmethylsulfonyl fluoride, 10 μg/ml aprotinin, 10 μg/ml leupeptin, pH 7.4, and 1% (v/v) Triton X-100. Lysates were clarified by centrifugation at 12,000g for 20 min at 4 C. The protein concentrations in the cell lysates were measured using a Bio-Rad DC (detergent compatible) assay. As already described [25,30], the same amount (50/80 μg of protein/lane) of proteins were denatured by boiling in Laemmli sample buffer containing 10% 2-mercaptoethanol. Proteins were separated by SDS-polyacrylamide gel electrophoresis and blotted on Immobilon-P membranes (Millipore, Billerica, MA, USA). Membranes were blocked for 1 h in TBS tween (10 mM Tris-HCl, pH 7.4, and 140 mM NaCl) containing 3% (w/v) bovine serum albumin and then incubated with the indicated antibodies.

Detection of blotted proteins was performed by ECL according to the manufacturer's instruction. Densitometric analysis was performed using Image Lab software 3.0 (Bio-Rad, Hercules, CA, USA). To evaluate insulin response, cells were serum starved for 24 h in media containing 0.25% BSA, with or without 1 nM BPA and then stimulated with 100 nM of insulin for 10 min.

### 2.5. Oil Red O staining

To measure cellular neutral lipid droplet accumulation, 3T3-L1 mature adipocytes were washed three times with iced phosphate-buffered saline (PBS) and fixed with 4% paraformaldehyde for 30 minutes. After fixation, cells were washed three times and stained with Oil Red O (ORO) solution (working solution 0.5 g ORO power dissolved in 60% ethanol) for 15 minutes at room temperature. Cells were washed again three times with PBS to remove unbound staining. 3T3-L1 mature adipocytes were examined under a light microscope and the red oil droplets stained in the cells indicate lipid accumulation. Two different microscopic fields (10X and 20X magnifications) per culture were photographed. The red oil droplets stained in the cells were extracted in 100% isopropanol. The absorbance was evaluated at 510nm as previously described [31].

### 2.6. Glucose utilization

For glucose utilization studies, the method previously described [32] was modified for 3T3-L1 adipocytes. Mature adipocytes were incubated for 24h in serum-free media containing 0.25% BSA, with or without 1 nM of BPA and stimulated with 100 nM of insulin. Glucose concentration was measured in the medium before and after the incubation. The difference in glucose concentration was considered to be utilized by the cells. Quantitative analysis of glucose concentration was performed with ABX Pentra 400 clinical chemistry analyzer using the reagent ABX Pentra Glucose CP (ABX-Horiba, Montpellier, France), according to the manufacturer’s instructions.

### 2.7. Statistical analysis

Data were analyzed with Statview software (Abacus concepts) by one-factor analysis of variance. Statistical analysis was conducted using Student's *t*-test for unpaired samples. *P* values of less than 0.05 were considered statistically significant. All values were expressed as means ± SD.

## 3. Results

### 3.1. BPA enhanced cell growth and expression of adipogenic and inflammatory markers

**Fig 1:**
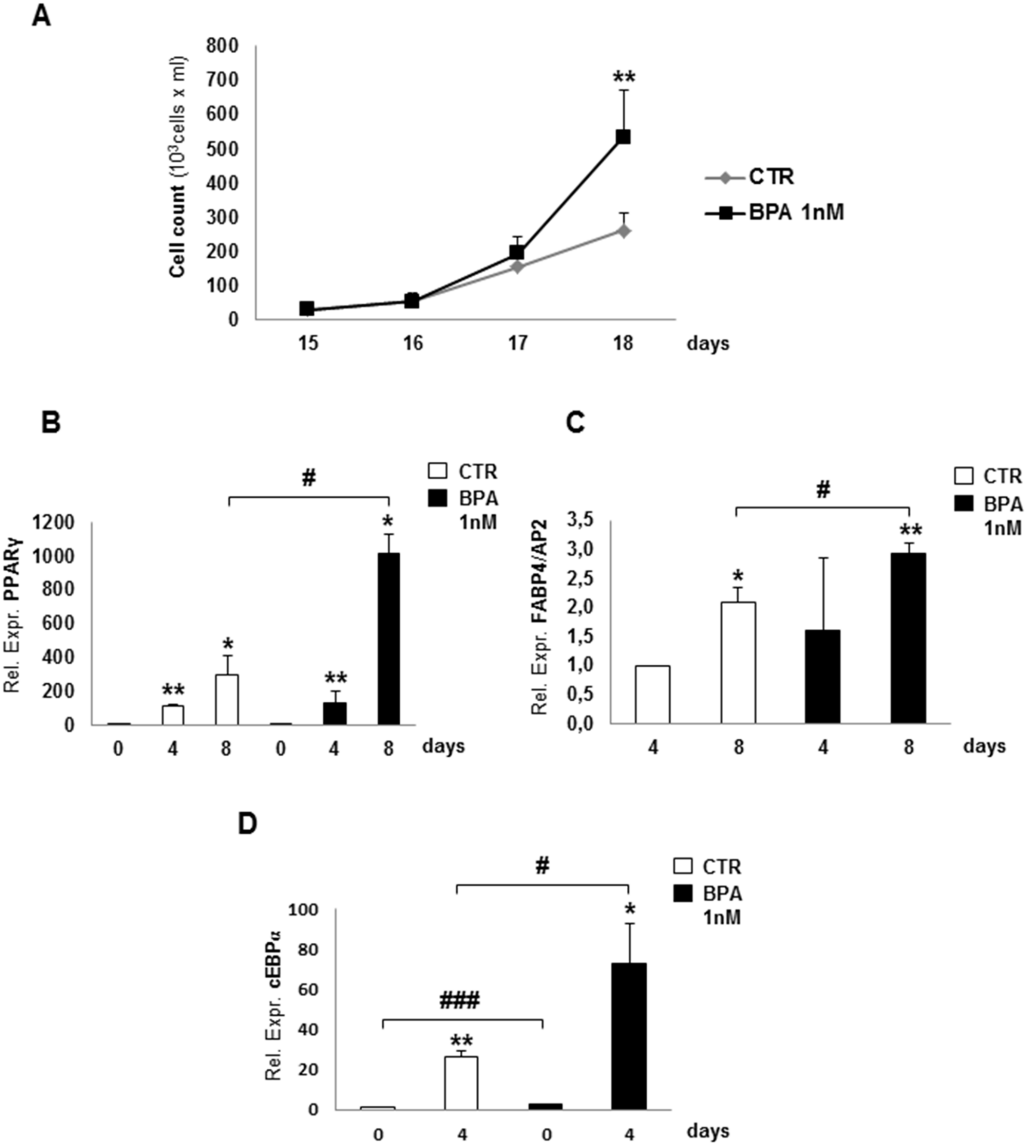
Effect of BPA on 3T3-L1 proliferation and mRNA gene expression. **(A)** 3T3-L1 pre-adipocyte were counted and expressed as cells/ml, at days 15, 16, 17 and 18, after 2 weeks of incubation with (BPA) or without (CTR) BPA 1nM, before adipogenesis started. PPARγ **(B)**, FABP4/AP2 **(C)** and cEBPα **(D)** mRNA levels were assayed during adipogenesis at days 0, 4 and 8, by Real-time RT-PCR analysis, expressed as Relative Expression Unit (REU). Bars represent the mean ± SD of four independent experiments. Asterisks indicate statistically significant differences (*p<0.05; **p<0.01; ***p<0.001) at days 4 and 8 compared to untreated day 0 for PPARγ **(B)**, at day 8 compared to untreated day 4 for FABP4/AP2 **(C)**, and at day 0 and day 4 compared to untreated day 0 for cEBPα **(D)**, without or with BPA incubation. Hashes (# p< 0.05; ### p<0.001) express statistically significant differences between day 8 with or without BPA incubation **(B and C)** and between day 0 and day 4 with or without BPA incubation **(D)**.

3T3-L1 mouse fibroblasts were grown in the absence or in the presence of BPA 1nM for three weeks before adipogenesis started. No relevant morphological abnormalities in 3T3-L1 pre-adipocytes were observed following BPA exposure. Interestingly, no significant difference in cell growth was observed up to day 15 (the end of the second week of treatment) in BPA treated cells compared to control cells. Thereafter, cells cultured with BPA showed a significant increase in number compared to untreated adipocytes (p*<*0.01) (Fig 1A), confirming its chronic effect on cell replication.

To evaluate BPA effects on adipocyte differentiation, mRNA and protein levels of the main adipogenic markers were assayed in BPA-treated and untreated 3T3-L1 cells. Following BPA exposure, both PPARγ and FABP4/AP2 mRNAs were significantly increased at day 8 from the start of the differentiation process, when compared to untreated cells (p<0.05) (Fig 1B and 1C). Notably, C/EBPα mRNA levels were increased significantly both at day 0 and day 4 of adipogenesis in differentiating 3T3-L1 cells treated with BPA compared to control cells (p<0.001 and p<0.05, respectively) (Fig 1D). We did not report C/EBPα mRNA levels at day 8 (the end of differentiation process), because it reaches a plateau after inducing the expression of PPARγ [33]. Moreover, BPA did not significantly affect Glucose Transporter 1 (GLUT-1) and GLUT-4 mRNA levels (data not shown).

**Fig 2:**
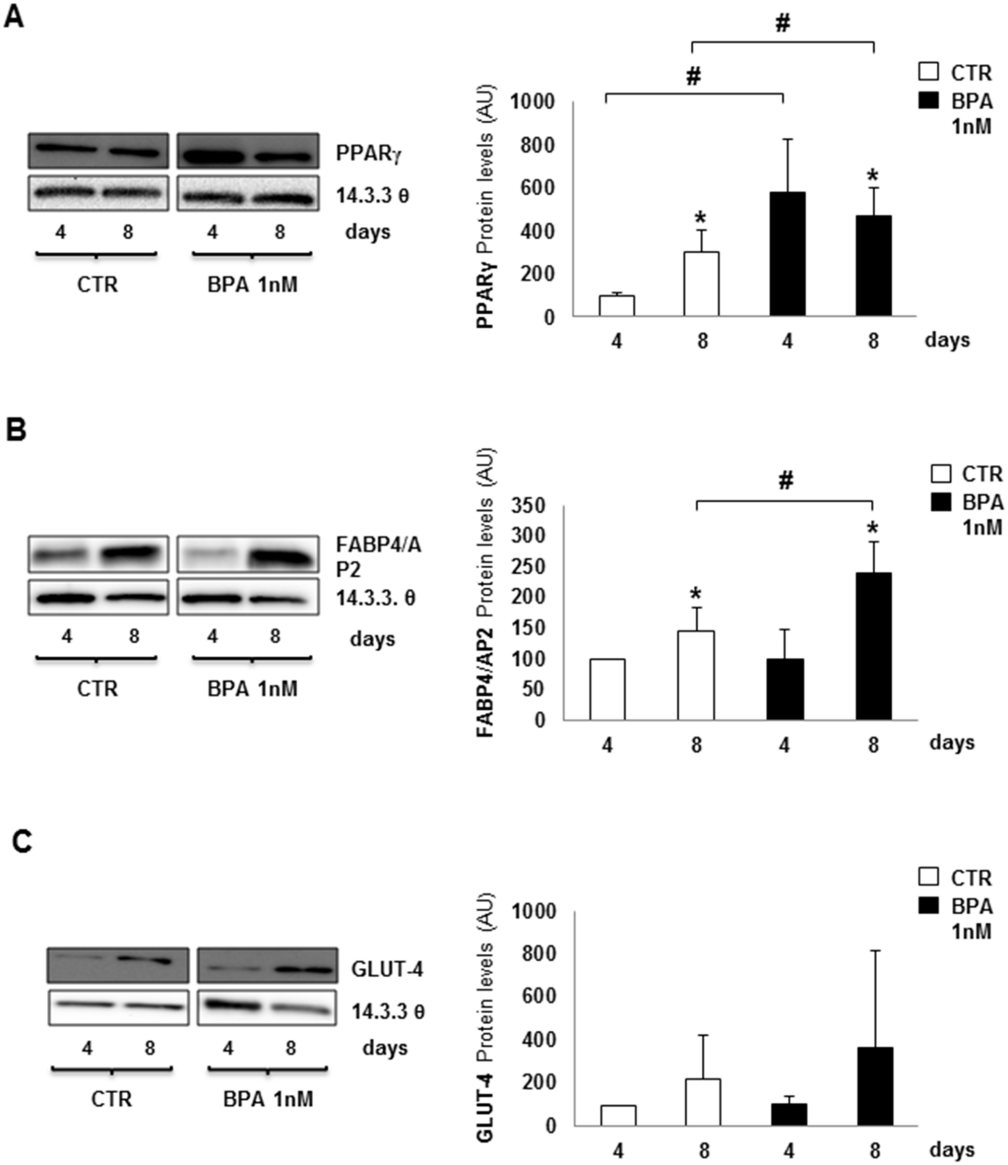
Effect of BPA on 3T3-L1 protein abundance of master differentiation genes. Protein levels of PPARγ **(A)**, FABP4/AP2 **(B)** and GLUT-4 **(C)** were assayed during adipogenesis at days 4 and 8 by western blot analysis, expressed as Arbitrary Unit (AU). Bars represent the mean ± SD of four independent experiments and blot is representative of four different experiments. Asterisks indicate statistically significant differences (*p<0.05) between days 4 and 8 for PPARγ **(A)** and day 8 for FABP4/AP2 **(B)**, without and with BPA incubation, both compared to untreated day 4. Hash (#p<0.05) expresses statistically significant differences between days 4 and 8 for PPARγ **(A)** and day 8 for FABP4/AP2 **(B)**, upon BPA incubation compared to controls. No significant differences in GLUT-4 protein expression **(C)**.

Similarly, in cells chronically incubated with BPA, PPARγ protein levels increased significantly both at day 4 and day 8 of adipogenesis (p<0.05) (Fig 2A), while FABP4/AP2 only at day 8 (p<0.05) (Fig 2B). Again, GLUT-4 protein abundance did not significantly change (Fig 2C). Interestingly, however, PPARγ protein abundance was already high at earlier days (data not shown).

**Fig 3:**
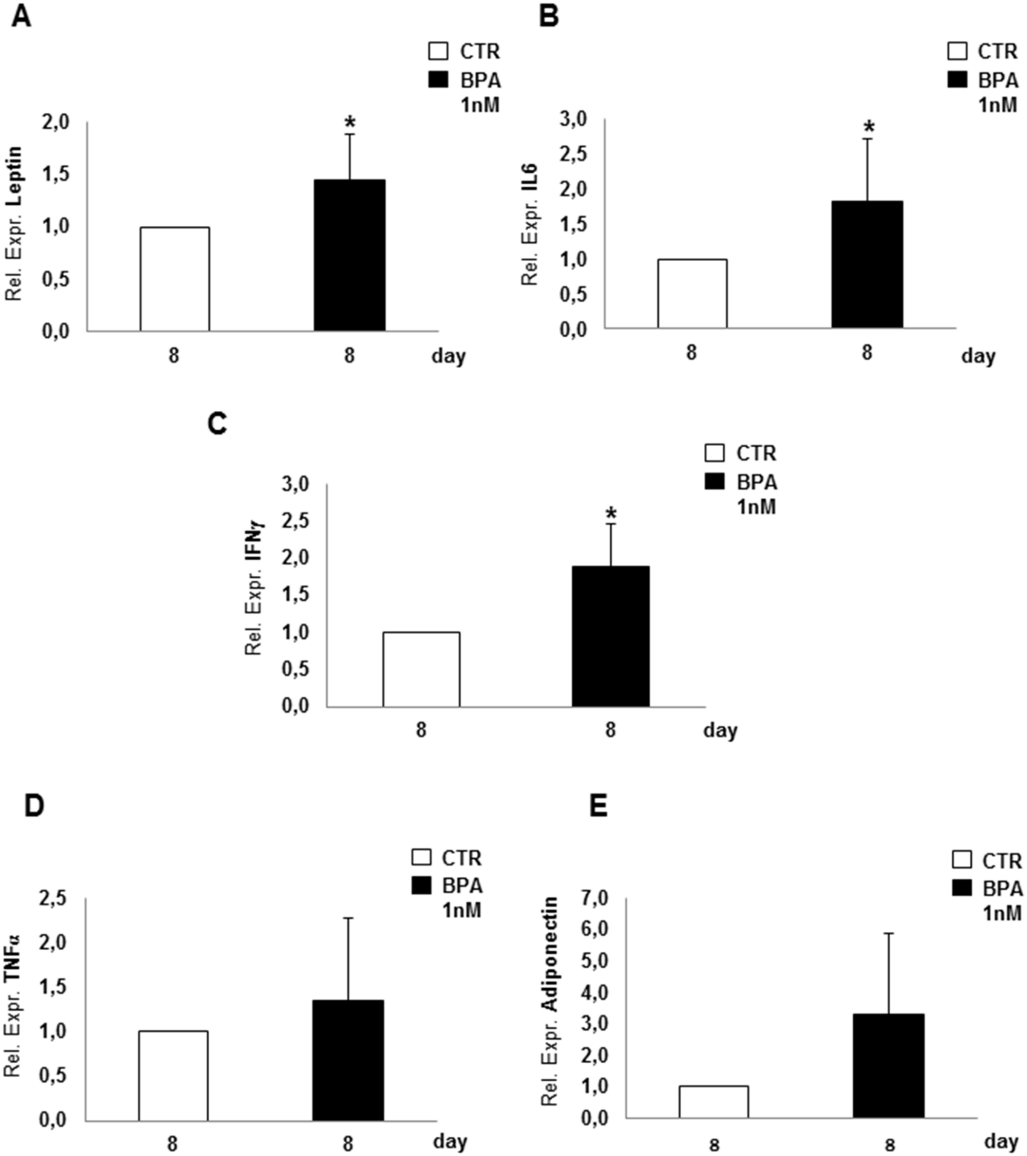
Pro-inflammatory effect in 3T3-L1 mature adipocytes. In mature adipocytes, mRNA levels of Leptin **(A)**, IL6 **(B)**, IFNγ **(C)**, TNFα **(D)** and adiponectin **(E)** were assayed at day 8, the end of adipogenesis, by Real-time RT-PCR analysis, and expressed as Relative Expression Unit (REU). Bars represent the mean ± SD of four independent experiments. Asterisk indicates statistically significant difference (*p<0.05) between adipocytes cultured upon BPA treatment compared to controls.

Next, we have investigated whether BPA may regulate adipocyte expression of adipokines and inflammatory factors. At the end of adipogenesis (day 8) Leptin (Fig 3A), IL6 (Fig 3B) and IFNγ (Fig 3C) mRNA levels displayed slight but significant increases upon BPA exposure (p<0.05), while no significant difference was observed in TNFα and adiponectin (adipoQ) expression (Fig 3D and 3E) in mature adipocytes.

### 3.2. BPA affected lipid accumulation, glucose utilization and insulin signalling

**Fig 4:**
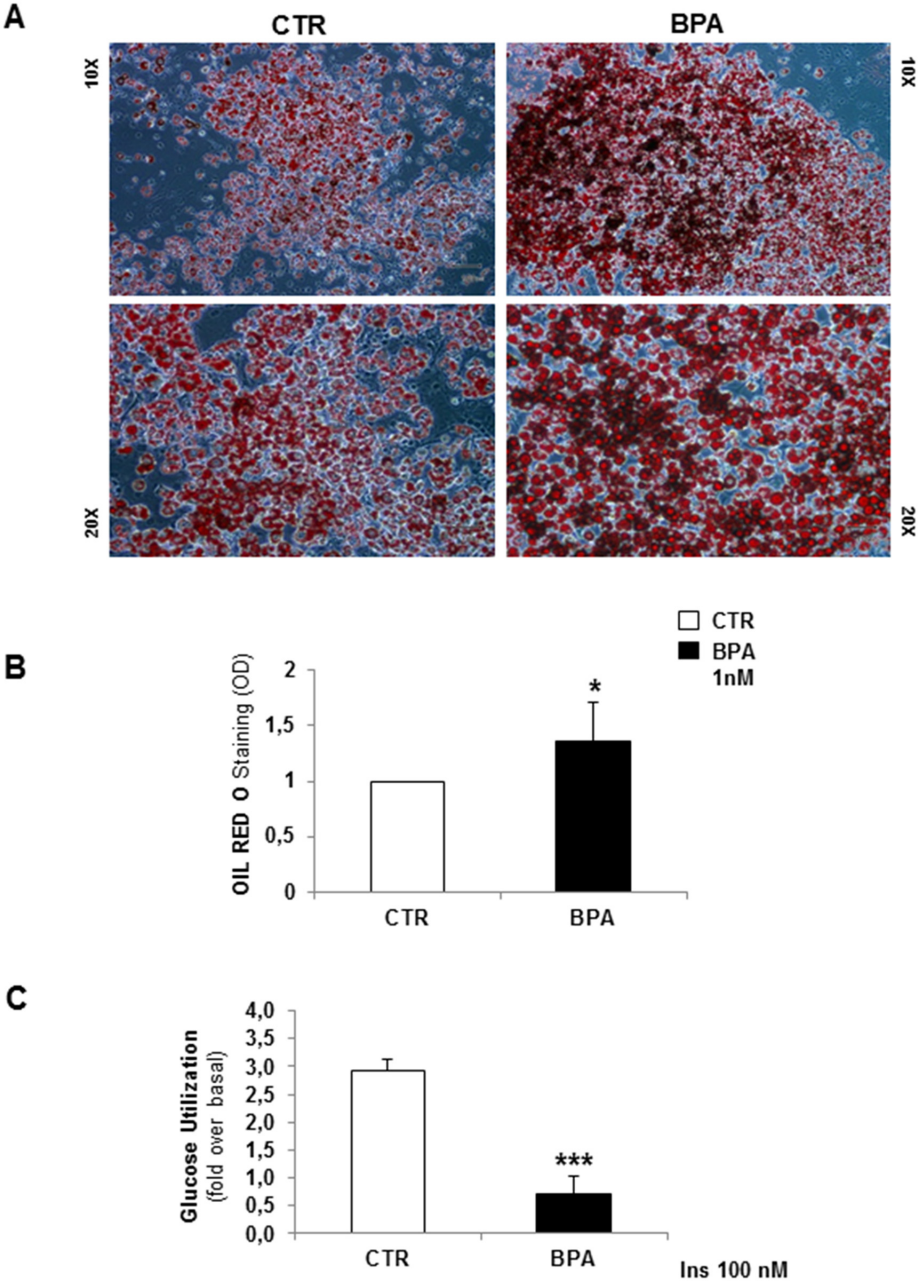
BPA effect on cellular neutral lipid droplet accumulation and glucose utilization in 3T3-L1 mature adipocytes. The microphotographs were obtained with an optical microscope in two original magnifications (10X and 20X), following ORO staining **(A)** in adipocytes upon BPA incubation compared to control cells. Lipid quantification test **(B)** was expressed as optical density (OD). Insulin stimulated glucose utilization test, expressed as fold over basal, was shown in differentiated 3T3-L1 cells incubated with 1 nM of BPA **(C)**. Bars represent the mean ± SD of four independent experiments. Asterisks indicate statistically significant differences (*p<0.05 and ***p<0.001) between adipocytes cultured upon BPA compared to controls.

**Fig 5:**
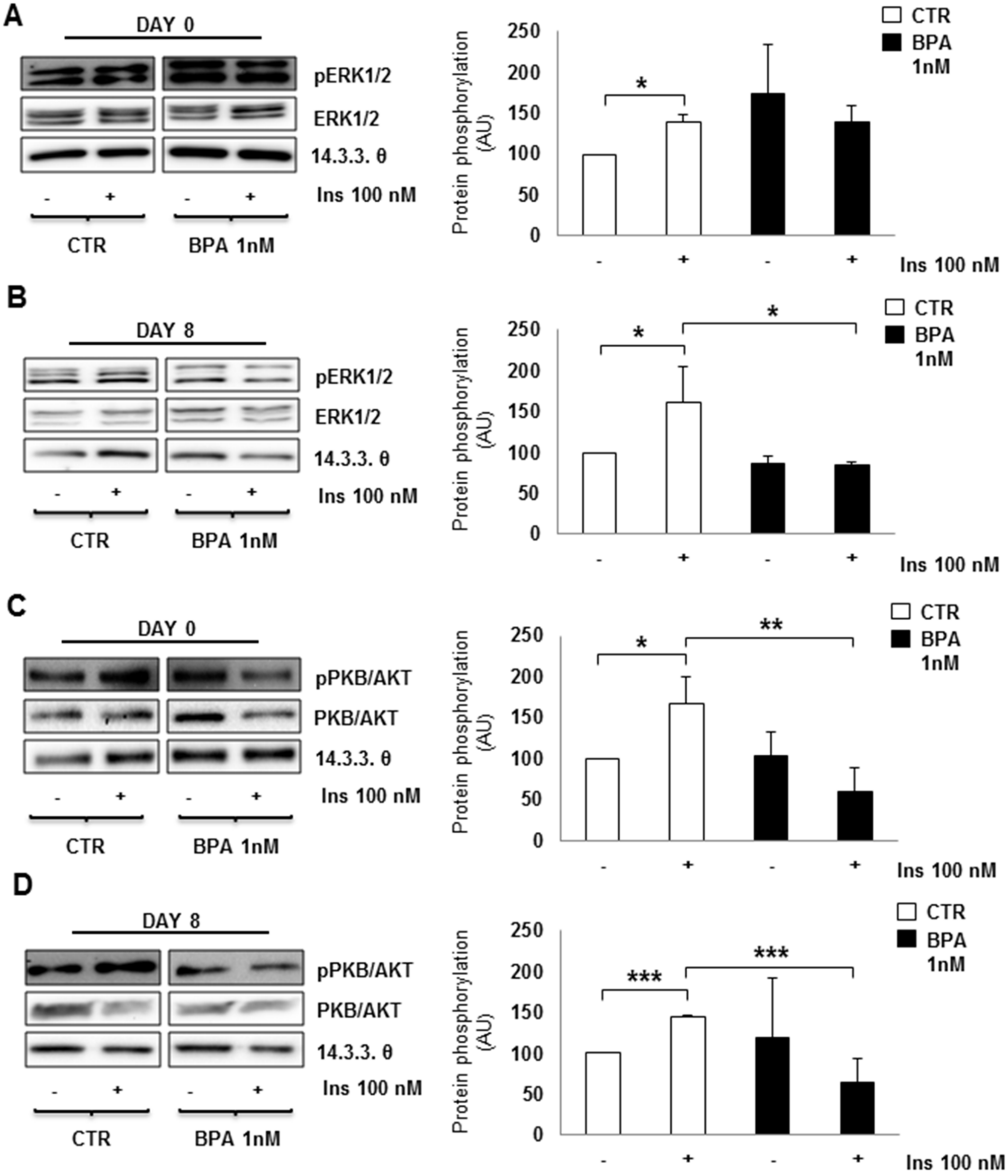
BPA effect on insulin transduction pathway. Insulin signaling was tested in 3T3-L1 pre-adipocytes, before differentiation started (day 0; **A-C**) and in mature adipocytes (day 8; **B-D**). ERK1/2 (**A-B**) and PKB/AKT (**C-D**) phosphorylation after insulin stimulation were shown in untreated and BPA treated cells. Bars represent the mean ± SD of four independent experiments and blot is representative of four different experiments. Asterisks indicate statistically significant difference (*p<0.05, **p<0.01 and ***p<0.001) in adipocytes cultured upon BPA compared to controls.

Fig 4A shows microphotographs of mature 3T3-L1 adipocytes stained with ORO. An increase in lipid droplet accumulation was evident in cells cultured with low and chronic BPA doses before and during adipogenesis process, compared to untreated cells. Data were confirmed by lipid quantification, showing a significant increase in lipid content for adipocytes cultured in presence of BPA (p<0.05) (Fig 4B), compared to control cells. Next, we have measured insulin stimulated glucose utilization in differentiated 3T3-L1 cells. Interestingly, BPA exposure caused significant >2-fold reduction of insulin-stimulated glucose utilization (p<0.001) (Fig 4C). To verify whether BPA may affect insulin signaling, we tested the ERK1/2 and PKB/AKT phosphorylation in 3T3-L1 pre-adipocytes (day 0) and mature adipocytes (day 8), after 10 min of insulin stimulation. In control cells, insulin exposure increased both ERK1/2 and PKB/AKT phosphorylation by 1.5 fold. Although other reports have detected much more robust response to insulin [34], the observed insulin-stimulated increases were statistically significant (p<0.05). At variance, in the presence of BPA, insulin failed to induce any increase of ERK1/2 (Fig 5A and 5B) and PKB/AKT (Fig 5C and 5D) phosphorylation. In particular, an inhibition of insulin effect was detected both in undifferentiated (day 0) (Fig 5A and 5C) and differentiated (day 8) (Fig 5B and 5D) 3T3-L1 cells. Interestingly, in BPA-treated cells, after insulin stimulation, PKB/AKT phosphorylation was significantly lower than in untreated cells, both at day 0 and 8 (p<0.01 and p<0.001, respectively), while ERK1/2 phosphorylation only at day 8 (p<0.05).

## 4. Discussion

Mounting evidence strongly suggests that BPA may affect adipose tissue development and function, supporting the “environmental obesogen hypothesis” [3,35]. In the current study, we have observed that prolonged exposure to low doses of BPA affected adipocyte differentiation program, by increasing pre-adipocyte growth and by altering master regulatory genes of adipogenesis. Indeed, a significant increase of PPARγ, FABP4/AP2 and C/EBPα expression was detected. Interestingly, when differentiation program was carried out in the presence of BPA, enhanced lipid accumulation in mature adipocytes was also observed. Moreover, mature adipocytes obtained upon BPA treatment showed a significant increase in pro-inflammatory cytokine expression (Leptin, IL6 and IFNγ), further supporting the BPA inflammatory effect that may contribute in the alteration of insulin sensitivity in the adipose tissue. As already reported in other in vitro studies [9,10], environmental exposure was mimicked by chronically treating cells with 1nM BPA (corresponding to 0.23 ng/ml) before and during differentiation. This BPA dose was chosen also based on dose-response experiments (not shown) indicating that lower doses (0.1 nM) were ineffective while higher doses were cytotoxic. Moreover, Schönfelder et al. and Padmanabhan V et al. [36,37] have reported a median BPA concentration in fetal and maternal blood of 22 ng/ml, even higher than 0.23 ng/ml (1nM).

We are aware that the limit of this research can be related to the lack of evidence of BPA detrimental effects on human adipogenesis. However, 3T3-L1 cells are commonly used to test the effects of several chemical compounds. Nevertheless, similar BPA interferences on insulin action and inflammatory pathways have been evidenced in murine and in human adipocytes [9,38–40].

Newsworthy, it has been recently postulated that metabolic diseases, including obesity and diabetes, may originate from developmental defects, even occurring in fetal life, that can be worsened by later environmental challenges [18,19,41–46]. Indeed, considering that fetal life is a critical window for the adipose tissue development, our data are in agreement with the hypothesis that the early chronic exposure to low BPA doses could be responsible for alteration in genes, involved in adipogenic commitment and adipocyte differentiation, with possible adult-onset of metabolic alterations. Thus, BPA may both enhance the expansion of adipocyte precursors and increase lipid content. Notably, hyperplastic obesity is related to the increased recruitment of new precursor cells, with features of metabolic “healthy” obesity, while the hypertrophic obesity is associated with abdominal obesity, ectopic fat accumulation, metabolic syndrome and genetic predisposition to diabetes [2]. Apparently, the significant increase in pre-adipocyte growth in presence of BPA may account for development of hyperplastic obesity. However, upon BPA incubation, we observed an increased lipid accumulation, with hyper-production of pro-inflammatory cytokines and generation of insulin resistant mature adipocytes. All these findings are reminiscent of hypertrophic obesity phenotype. In this scenario we can hypothesize that BPA, through a precocious interference, can increase both adipocyte number and lipid content, since it is capable to affect pre-adipocytic cell growth and to alter timing and expression of master genes involved in adipocyte differentiation and adipose tissue development. Subsequently, BPA may generate metabolic dysfunctional 3T3-L1 adipocytes, with insulin resistance, as indicated by down-regulation of insulin signalling and reduction of glucose utilization. Finally, the pro-inflammatory action exerted by BPA may worsen insulin sensitivity, as we have previously reported in human adipocytes acutely treated with low BPA doses [9].

Our observations are in line with the enhanced effect of BPA on adipocyte differentiation, which has been recently reported [5,37,38,47,48]. Indeed, in our experiments, BPA was added to the culture media before differentiation started, to study its chronic effect and to better mimic the impact of early environmental exposure on undifferentiated cells. Remarkably, at the earlier time of pre-adipocytes commitment, the effects of BPA exposure at doses within the range of those found in biological fluids are in agreement with the concept that the timing of pollutant exposure is a key factor to develop dysfunctional adipose tissue [10,11].

Considering BPA widespread distribution and human timing of exposure (i.e. prenatal/postnatal periods), in this paper we have reinforced the hypothesis that BPA, by acting during developmental period, may build up dysfunctional adipose tissue, responsible for alteration in programming the adult body weight and in predisposition to overweight and visceral obesity in a sex- and diet-dependent manner. In this regard, BPA could be more deleterious in humans if associated with a concomitant exposure to other environmental chemical compounds, resulting as a “cocktail effect” [6,49].

Interestingly, this study pointed the attention on PPARγ, FABP4/AP2 and C/EBPα, nuclear receptors with a central role in regulating energy homeostasis and essential for the normal physiological function of most mammalian cell types, tissues and organs. In particular, PPARγ acts as major regulator for adipogenesis and lipid metabolism and it has been used as proxy bio-marker for screening of environmental obesogens [49]. The mechanism by which BPA affects these genes is still unknown and will be object of further researches, although genetic, epigenetic and endocrine disruption mechanisms, with the possible involvement of oxidative stress, mitochondrial dysfunction and cell signaling have been hypothesized [46,50,51].

It should also be noticed that fetal/neonatal and childhood liver has poor capacity to inactivate BPA via conjugation, leading to relatively higher free BPA urine and plasma concentrations in toddler compared to adults [14–16]. Interestingly, placenta is able to transfer BPA-glucuronide that can be deconjugated in fetus, with subsequent higher BPA concentrations in fetal amniotic fluid, higher fetal exposure and alteration in adipose tissue development. The observation that in early childhood daily BPA intake is related to different non-dietary sources exposure, such as dermal sublingual and inhalational exposures, with increased free BPA levels [40,52,53] could also be relevant.

However, adipose tissue turnover also occurs in adult life [22]. Therefore, the disrupting effect of prolonged BPA exposure cannot be excluded even in adult adipose tissue.

In this context, our data strengthened the concept that BPA may alter genes involved in adipogenesis, particularly during embryonic/fetal life when fat tissue develops and when commitment of pre-adipocytes to the adipogenic lineage occurs. As consequence, alteration in adipose tissue function, in insulin sensitivity and fat accumulation, may occur not only via well documented estrogen-mediated processes, but also through pathways that remain to be determined, especially in humans.

Certainly, a proper development of adipose tissue and adipose tissue function are critical factors for regulation of fat mass as well as for glucose homeostasis late in life.

In conclusion, this study has evidenced that when 3T3-L1 pre-adipocytes were chronically cultured in presence of BPA, alterations in adipocyte differentiation occurred. BPA exposure could contribute to hyperplastic and hypertrophic obesity, the first related to increased cell number, the second to generation of impaired adipocytes, capable to accumulate more lipids, to be less insulin sensitive and to secrete more pro-inflammatory cytokines.

Further studies are needed to investigate BPA effects on human adipose tissue and to better define its role in human metabolic health, together with the mechanisms involved. In fact, understanding BPA mechanisms responsible for impaired adipogenesis may offer novel ways to promote public health and avoid metabolic consequences of obesity. In the mean time, to reduce BPA environmental chronic exposure would be beneficial to protect vulnerable individuals, such as pregnant women, infants and children and to prevent metabolic dysfunctions and negative outcomes later in the offspring life.
